# Mediterranean Diet and Physical Activity as Healthy Lifestyles for Human Health

**DOI:** 10.3390/nu14122514

**Published:** 2022-06-17

**Authors:** Daniela Bonofiglio

**Affiliations:** 1 Department of Pharmacy, Health and Nutritional Sciences, University of Calabria, 87036 Rende, Cosenza, Italy; daniela.bonofiglio@unical.it; Tel.: +39-0984-496208; Fax: +39-0984-496203; 2 Centro Sanitario, University of Calabria, 87036 Rende, Cosenza, Italy

Health status is influenced by several factors, such as proper dietary pattern and regular physical activity (PA), which are crucial elements of lifestyle in terms of the prevention and treatment of metabolic and chronic diseases in all stages of life and particularly during childhood and adolescence.

In the last decades, cultural globalization and urbanisation have led to the “Westernization” of lifestyles, characterized by the increased consumption of foods with high quantities of refined carbohydrates, sugars, salt, saturated fats, proteins, as well as poor fruits and vegetables, and by increasing sedentariness. This phenomenon has generated a “nutritional transition” whereby obesity and diet-related chronic diseases represent new challenges for public-health systems. In contrast to this global trend and in the context of healthy eating habits, the dietary pattern inspired by Mediterranean Diet (MD) principles is associated with multiple health benefits, due to its protective effects against a wide range of chronic and metabolic diseases, including obesity, diabetes mellitus, cardiovascular and neurodegeneretive diseases, and cancers [1,2,3]. The traditional MD, usually consumed among the populations bordering the Mediterranean Sea many years ago, has entered the medical literature following publications of Seven Countries Study’s results by the legendary Ancel Keys [4]. The MD pattern is characterized by high intakes of vegetables, legumes, fruits and nuts, cereals (that in the past were largely unrefined), and dairy products; high consumption of extra virgin olive oil; low intakes of saturated lipids; moderately high intakes of fish and poultry; low intakes of meat and sweets; and regular but moderate intake of wine generally during meals [5]. Seasonality, biodiversity, traditional and local food products are also important components in this eating model. In addition, the MD has qualitative cultural and lifestyle elements, such as conviviality, culinary activities, adequate rest, and physical activity [5]. Regarding PA, the WHO (World Health Organisation) recommends performing moderate-intensity levels of PA for ≥150 min/week, and vigorous-intensity levels of PA for ≥2 days/week to have health benefits [6]. Overall, the MD, including PA as an integral part of the traditional Mediterranean lifestyle, is also a universal, cultural, social and spatial heritage of all civilizations living around the Mediterranean basin registered by the UNESCO (United Nations Educational, Scientific and Cultural Organization) as an immaterial human heritage in 2010. Despite its increasing popularity worldwide, adherence to the MD model is decreasing due to multifactorial influences determining the ongoing erosion of this dietary pattern and cultural heritage worldwide, even in the Mediterranean area. Thus, the need to investigate the current dietary habits and to increase population awareness of the importance of MD pattern is becoming urgent.

The Special Issue of Nutrients entitled “Mediterranean Diet and Physical Activity as Healthy Lifestyles for Human Health” was devoted to collecting original research and reviews of literature concerning the adherence to the MD and various health outcomes. New information has been added in this field by means of ten articles, with nine original papers and one narrative review.

A widely considered aspect was the evaluation of the MD adherence in association with PA in different target populations, such as children, adolescents, and adults, as well as populations from different geographical location worldwide.

Firstly, Ceraudo et al. [7], evaluating the impact of the Mediterranean food choices on serum metabolic profile in healthy adolescents performing different intensity levels of PA, showed that active subjects who consume typical Mediterranean foods had a better serum metabolic profile, suggesting that adhering to the MD pattern and performing PA led to a significant reduction in glucose and lipid profile, thus making the MD and PA a winning combination for health status.

A comparative study carried out among 2722 individuals aged 2 to 24 years living in Croatia showed low adherence to the MD over the entire sample [8]. Specifically, Matana et al., found that the prevalence rate of poor adherence to the MD increased with higher education stage, while the lowest rate was observed for the children enrolled in kindergartens [8]. In agreement with other studies conducted in the same age range [9,10], individuals physically active were also higher adherent to the MD, fostering the positive association of both factors with healthy lifestyle habits.

The association between PA and adherence to the MD was also investigated in 1220 fitness-center users in Croatia [11]. Results showed that MD adherence, measured by means of the Mediterranean Diet Serving Score (MDSS) in the whole study sample (mean age was 29.1 ± 8.8 years) was 8.0, and 18.6% of participants adhered to the MD (total MDSS score ≥ 14). Interestingly, there was a significant positive correlation between the level of PA and the MDSS score in this population, without gender differences. Thus, from these findings it is possible to speculate that promoting adherence to the MD along with PA guidelines might provide a more comprehensive endorsement to obtain greater health benefits, over and above those acquired separately by the MD and PA.

Another Croatian group of research [12] found that MD adherence, evaluated by MDSS, had a prevalence of 28.5% in 4671 adult subjects, with higher odds in women, older subjects, and people with a higher level of objective material status. Over the study period, the absolute change in the MD score positively associated with female gender, age, higher education, and moderate physical activity, but it was negatively correlated with adherence to the MD at baseline, suggesting that these factors can be potentially targeted in order to increase MD adherence.

Among three other studies carried out in both Mediterranean [13,14] and non-Mediterranean countries [15], the first involved 1512 Spanish adults, aged 55–80 years, with overweight/obesity and metabolic syndrome. In this cross-sectional study, analyzing data from a sub-sample of the PREDIMED-Plus study, socioeconomic status was related to an unhealthy dietary pattern associated with low PA [13]. This indicates that community interventions and health policy decisions may target subsets of the population in order to promote a healthier lifestyle. The second one [14] investigated in an Italian adult sample the relationship between adherence to the MD with the self-perceived adoption of a sustainable diet, as well as demographic, socioeconomic, and behavioral variables. Results showed that MD scores were high in female subjects, in subjects with higher income and educational level, in subject who consider the MD a sustainable dietary model, as well as those who perceive themselves as following a sustainable diet. Globally, a medium adherence to the MD was found [14], in line with another recent investigation on an Italian adult population [16]. Intriguingly, eating pattern, perceived knowledge, and benefits have been evaluated by Cuoto et al. [15] in a Portuguese immigrant community living in California. Even though Portugal is geographically not in the Mediterranean basin, the MD is a settled cultural heritage of the Portuguese population. MD scores were higher in adults from the immigrant than in those from the American population, and the perceived health benefits of the diet was a key factor in adherence to the MD only in the Portuguese immigrant community. The authors reported the need for further investigations to confirm these results.

Apart from the studies evaluating adherence to the MD in different contexts, also interesting was the relationship between diet and some metabolic and chronic diseases, such as type 1 diabetes (T1D) and cancer. In this scenario, Antoniotti et al. [17] assessed the MD adherence by the Mediterranean Diet Quality Index (KIDMED) questionnaire in 65 children and adolescents with T1D in relation to metabolic control. KIDMED scores displayed average values in 58.6% of the subjects, in line with data from a non-diabetic population of Italians of the same age range [8]. Low adherence to the MD was associated with a high risk of obesity in T1D, as reported in the general pediatric and adult population [17]. Regarding the prospective relation between diet pattern and total cancer risk, Yiannakou et al. [18] examined in a prospective study the longitudinal association between the Mediterranean-style dietary pattern (MSDP) score, derived from a semi-quantitative food frequency questionnaire, and total cancer risk in 2966 participants of the Framingham Offspring Study cohort, displaying weaker association between MSDP score and cancer risk in men except in non-smokers, while higher adherence to MSDP was associated with lower cancer risk, especially among women [18], confirming that the MD as a source of bioactive compounds protects against cancer [19].

Last, the impact of geographical location on MD adherence has been also considered and discussed in a narrative review by Mattavelli et al. [20] who highlighted the relevance of geographical location and related social features in the current moderate adherence to the MD in adolescents as well as in adults, fostering awareness that will lead to the promotion of the MD as a global nutritionally balanced and healthy dietary pattern even in the countries of its origin.

Overall, all studies included in this Special Issue provide an update on the MD adherence in the population at different age stages and from different countries, highlighting some opportunities and challenges for the adoption of an MD eating pattern along with PA to reduce metabolic and chronic disease risk and to obtain greater health benefits.
